# Rapid and Scalable Plant-based Production of a Cholera Toxin B Subunit Variant to Aid in Mass Vaccination against Cholera Outbreaks

**DOI:** 10.1371/journal.pntd.0002046

**Published:** 2013-03-07

**Authors:** Krystal Teasley Hamorsky, J. Calvin Kouokam, Lauren J. Bennett, Keegan J. Baldauf, Hiroyuki Kajiura, Kazuhito Fujiyama, Nobuyuki Matoba

**Affiliations:** 1 Owensboro Cancer Research Program, Owensboro, Kentucky, United States of America; 2 Department of Pharmacology and Toxicology and James Graham Brown Cancer Center, University of Louisville School of Medicine, Louisville, Kentucky, United States of America; 3 International Center for Biotechnology, Osaka University, Osaka, Japan; Federal University of São Paulo, Brazil

## Abstract

**Introduction:**

Cholera toxin B subunit (CTB) is a component of an internationally licensed oral cholera vaccine. The protein induces neutralizing antibodies against the holotoxin, the virulence factor responsible for severe diarrhea. A field clinical trial has suggested that the addition of CTB to killed whole-cell bacteria provides superior short-term protection to whole-cell-only vaccines; however, challenges in CTB biomanufacturing (i.e., cost and scale) hamper its implementation to mass vaccination in developing countries. To provide a potential solution to this issue, we developed a rapid, robust, and scalable CTB production system in plants.

**Methodology/Principal Findings:**

In a preliminary study of expressing original CTB in transgenic *Nicotiana benthamiana*, the protein was *N*-glycosylated with plant-specific glycans. Thus, an aglycosylated CTB variant (pCTB) was created and overexpressed via a plant virus vector. Upon additional transgene engineering for retention in the endoplasmic reticulum and optimization of a secretory signal, the yield of pCTB was dramatically improved, reaching >1 g per kg of fresh leaf material. The protein was efficiently purified by simple two-step chromatography. The GM1-ganglioside binding capacity and conformational stability of pCTB were virtually identical to the bacteria-derived original B subunit, as demonstrated in competitive enzyme-linked immunosorbent assay, surface plasmon resonance, and fluorescence-based thermal shift assay. Mammalian cell surface-binding was corroborated by immunofluorescence and flow cytometry. pCTB exhibited strong oral immunogenicity in mice, inducing significant levels of CTB-specific intestinal antibodies that persisted over 6 months. Moreover, these antibodies effectively neutralized the cholera holotoxin *in vitro*.

**Conclusions/Significance:**

Taken together, these results demonstrated that pCTB has robust producibility in *Nicotiana* plants and retains most, if not all, of major biological activities of the original protein. This rapid and easily scalable system may enable the implementation of pCTB to mass vaccination against outbreaks, thereby providing better protection of high-risk populations in developing countries.

## Author Summary

Cholera sporadically causes outbreaks in regions where safe water supply and sanitation systems are not sufficient. As currently available vaccines are only effective for 2 to 3 years, reactive mass vaccination has been proposed to reduce mortality during outbreaks. Cholera toxin B subunit (CTB), when combined with killed whole-cell bacteria, has been shown to provide superior short-term protection, but manufacturing challenges of the protein limit its availability for mass vaccination programs in developing countries. Our work presented herein developed a rapid, robust, and scalable bioproduction system in plants for a CTB variant, pCTB. The system allowed for the accumulation of pCTB at >1 g per kg of fresh leaf of tobacco-related plants within 5 days, which accounts for over 1000 doses of original CTB included in the World Health Organization-prequalified vaccine Dukoral. We further analyzed in depth the integrity of pCTB using a series of biochemical, biophysical, and immunological experiments, demonstrating that the plant-made protein is feasible as a cholera vaccine antigen. Thus, pCTB plus killed bacteria may be ideal for reactive vaccination against cholera outbreaks.

## Introduction

Cholera is an acute watery diarrheal disease caused by the 01 and 0139 serogroups of *Vibrio cholerae*. In 2011 the World Health Organization (WHO) reported 589,854 cholera cases and 7816 deaths, revealing a 85% increase in the number of cases compared to 2010 [1]. The increase in outbreaks globally, has sparked debate on the use of mass cholera vaccination, a cholera vaccine stockpile, and use of reactive vaccination strategies. Currently, there are two WHO-prequalified oral cholera vaccines, Dukoral, Crucell and Shanchol, Shantha Biotechincs. Dukoral contains bacterially produced recombinant cholera toxin B subunit (CTB) plus killed whole-cells (WC) of *V. cholerae*, and has been found to be safe and effective over 25 years [2], [3], [4], [5], [6]. CTB is the non-toxic subunit of cholera holotoxin. The protein forms stable homopentamers with a molecular mass of about 55 kDa [7]. The CTB component of the vaccine induces holotoxin-neutralizing antibodies in the gut [8], which act synergistically with the anti-bacterial immunity in protection against cholera [9].

Due to the limited production capacity of fermentation tanks and the high manufacturing cost of CTB (Dukoral costs ∼U.S.$6 per dose to manufacture whereas Shanchol is manufactured at less than $2 per dose [10], [11]), much interest has been targeted towards the WC-only vaccine Shanchol, which is less expensive than Dukoral and moderately effective [12]. However, in a randomized double blind field trial in Bangladesh the addition of CTB to WC provided superior short-term protection to the WC-only group especially in children, i.e. 85% versus 58% protective efficacy in the first six months after vaccination. Furthermore, CTB-WC vaccine is cross protective against the heat-labile toxin-producing enterotoxigenic *Escherichia coli* (LT-ETEC). A large-scale field trial found that there were 67% fewer episodes of LT-ETEC in the CTB-WC group than in the WC-only group [13].

Both of the above vaccines, however, are only effective for 2 to 3 years [14], [15]. As such, recent studies have pointed to the significant value of reactive or delayed vaccine use [10]. In Vietnam, a case-control study found a protective efficacy of 76% with the reactive use of killed oral vaccines [16]. Using existing data from cholera outbreaks, simulations found that if widespread vaccination had been implemented during epidemics over the last decade, 40% of cases and deaths would have been prevented [17]. Furthermore, a firm consensus was reached by the WHO that cholera vaccines should be used reactively as an additional control measure for the management of cholera outbreaks [1]. Given CTB's capacity to induce neutralizing antibodies against the virulence factor responsible for diarrhea and to increase short-term protection, it may be ideal for CTB-WC vaccines to be used in reactive vaccination against outbreaks.

A number of different expression systems have been explored for the recombinant production of CTB and CTB fusion proteins. These include prokaryotic cells such as genetically modified *Vibrio cholerae* [18], *Escherichia coli* [19], and *Bacillus* and *Lactobacillus* spp. [20], [21], [22], as well as eukaryotes ranging from yeast cells [23] to the multicellular organisms such as silkworms [24] and plants [25], [26], [27], [28], [29]. Previously, we expressed a candidate HIV-1 vaccine based on a viral glycoprotein gp41's membrane proximal region peptide fused to the C-terminus of CTB (CTB-MPR) in transgenic *Nicotiana benthamiana* [30]. Interestingly, the CTB fusion protein had an *N*-linked glycan as CTB has a sequon (Asn-Ile-Thr) at position 4–6 (amino acid numbering based on the crystalized mature protein; Protein Database ID: 3CHB). Despite this unique modification not present in original *V. cholerae*-derived CTB, the plant-expressed protein retained pentameric structure with apparent nanomolar GM1-ganglioside binding affinity and was capable of inducing a gp41-specific antibody response in mice. As sequon-based protein *N*-glycosylation is conserved among eukaryotes [31], [32], recombinant CTB expressed in any eukaryotic system may be *N*-glycosylated. In fact, Miyata *et al.* recently showed that CTB expressed in *Pichia pastoris* was *N*-glycosylated at the Asn4 position [23]. However, other reports on eukaryotic systems that claimed to be a CTB production platform, most notably of transgenic plants such as potato [26], rice [29], tomato [33] and tobacco [34], [35], have addressed this issue with no or limited details. In addition, these plant-produced CTB molecules were not purified, and therefore it remains unknown if they can meet quality standards (identity, purity, potency and safety) required from regulatory agencies for pharmaceuticals.

Although plant-based expression systems hold the potential advantages of scalability and cost effectiveness compared with conventional cell culture-based systems of both prokaryotic and eukaryotic origins, modification with plant-specific *N*-glycans could pose potential concern(s) in terms of efficacy and/or safety. For the latter issue in particular, there has been a theoretical concern of allergenicity, given that many plant-derived allergens are glycoproteins [36]. Meanwhile, *N*-glycosylation of CTB raises another question of whether such a modification may actually play an important role in the protein's accumulation when expressed via the endomembrane system of eukaryotic cells, as *N*-glycans are known to help fold and stabilize proteins in the endoplasmic reticulum (ER) [32]. These questions can be simultaneously addressed if a non-glycosylated form of CTB is produced.

In this report, we confirmed that CTB expressed in transgenic *N. benthamiana* is indeed *N*-glycosylated, and provided a detailed glycan profile demonstrating the presence of plant-specific glycans. We then engineered an aglycosylated variant of CTB, for which a robust virus vector-based large-scale production system was successfully established. Furthermore, detailed biochemical and immunological analyses were performed to demonstrate the physicochemical stability, GM1-ganglioside-binding affinity, and vaccine efficacy of the aglycosylated plant-made CTB (pCTB).

## Methods

### Ethics statement

All experimental animal procedures described herein were approved by the University of Louisville's Institutional Animal Care and Use Committee (Protocol number: 12006). The maintenance and care of experimental animals complied with the University of Louisville Animal Care and Use Program's guidelines for the humane use of laboratory animals, which adhere to the National Research Council Guide for the Care and Use of Laboratory Animals, the NIH Public Health Service Policy on Humane Care and Use of Laboratory Animals, and the USDA Animal Welfare Regulations promulgated by the Animal Welfare Act.

### Construction of transgenic *N. benthamiana* expressing CTB

Transgenic *N. benthamiana* plants were created as previously described [30], using *Agrobacterium tumefaciens* LBA4404 harboring a pGPTV-kan vector containing the plant-expression-optimized synthetic *V. cholerae CTB* coding sequence (GenBank accession no. AY475128) with an 18-nucleotide extension (TCCGAGAAGGATGAACTC) at the 3′ end that encodes the C-terminal SEKDEL sequence for ER retention [25], [30].

### Analysis of *N*-glycans attached to transgenic *N. benthamiana*-expressed CTB

Transgenic *Nicotiana*-expressed CTB and the *Escherichia coli*-produced counterpart (see below) were purified as described below and analyzed by sodium dodecyl sulfate polyacrylamide gel electrophoresis (SDS-PAGE). After electrophoresis, proteins were transferred to a poly(vinylidene difluoride) membrane. Blots were probed with goat anti-CTB antiserum (List Biological Laboratories) followed by horseradish peroxidase (HRP)-conjugated rabbit anti-goat antibodies (Sigma-Aldrich), with 1.5 µg/ml concanavalin A (ConA)-HRP conjugate (Sigma-Aldrich), or with rabbit anti-peroxidase (Sigma-Aldrich) followed by HRP-conjugated goat anti-rabbit (Santa Cruz Biotechnology) antibodies. Antibodies were detected using chemiluminescence (ECL Prime; GE Healthcare) and images were acquired on a Kodak Image Station 4000R Pro. Glycan profiling was performed as previously described [30]. The glycans were released from CTB by hydrazinolysis, which were pyridylaminated and separated by reversed phase high-performance liquid chromatography (RP-HPLC) and size-fractionation (SF)-HPLC. The glycan structures were determined by mass spectrometry (MS) and/or by comparing their elution profiles in RP-HPLC with commercial 2-aminopyridine (PA)-labeled standards of known isomeric configurations.

### Vector construction

A “deconstructed” tobamovirus replicon system [37], [38] (magnICON; Icon Genetics GmbH) was used to express CTB in *N. benthamiana*. The *CTB-SEKDEL* coding sequence used for transgenic plant construction was sub-cloned into the magnICON vector pICH11599 to generate pNM47. A standard PCR method was used to remove the *V. cholerae* secretory signal from the original *CTB* gene, using pNM47 as a template. The resulting PCR product was sub-cloned into pIHC11599 to generate pNM134. Site directed mutagenesis was performed according to the manufacturer's instructions (Quikchange II Site-Directed Mutagenesis Kit; Agilent Technologies) using pNM134 as the template and primers that mutated the nucleotide A at position 74 (GenBank accession no. AY475128) to a G creating pNM156 (for Asn4→Ser CTB). For expression of pCTB with different secretory signal peptides, pNM156 was used as a template for PCR. An oligonucleotide corresponding to an appropriate secretory signal sequence (Table S1; *N. benthamiana* codon optimized) flanking the 5′ coding region of the *CTB* gene and an oligonucleotide corresponding to the 3′ region of the *CTB* gene+SEKDEL were used to amplify the secretory signal+CTB+SEKDEL-coding sequence. The resulting PCR products were sub-cloned into pIHC11599 to generate pNM226, 227, 228, 229, 230, 231, 232, and 257, respectively. For expression of pCTB with secretory signals other than the above mentioned sequences, the 5′ provectors pICH20155, pICH20188, pICH20388, and pICH20999 (Table S1) were used.

### Viral vector-based overexpression of CTB in *N. benthamiana*

Plant expression of the pCTB was performed using the magnICON system. For expression of pCTB with a secretory signal attached to the 5′ end of CTB, the plasmids pNM226, 227, 228, 229, 230, 231, 232, and 257 were used with pICH20111 and pICH14011. For CTB with the other secretory signals the appropriate 5′ provector (see above; Table S1) was used with pNM156 and pICH14011. The vectors were delivered into *N. benthamiana* leaves using the *Agrobacterium* vacuum infiltration method [39]. After 4–6 days, leaf material was homogenized by a Waring blender in extraction buffer (20 mM Tris-Cl, pH 5.0, 500 mM NaCl, 20 mM ascorbic acid, 10 mM sodium metabisulfite) and the extract was filtered through cheese cloth and miracloth. The extract was warmed to 50°C for 25 min and centrifuged at 22,100×g for 15 min at 4°C followed by filtration through a 0.22 µm filter. The clarified extract was analyzed for CTB expression by SDS-PAGE and GM1-ganglioside-capture enzyme-linked immunosorbent assay (GM1-ELISA) as described previously [25], [30], [40], [41].

### Expression of CTB in *E. coli* (eCTB)

The *V. cholerae CTB* gene (GenBank accession no. AAC34728) was cloned into pET-22b(+) (Novagen). This plasmid was transformed into electrocompetent BL21(DE3) cells. The bacterial cells were grown to an OD600 of ≈0.6. IPTG was added to a final concentration of 400 mM and the cells were cultured for 4 h. The supernatant containing CTB was separated from the cells by centrifugation at 22,100×g for 15 min at 4°C, and used for purification.

### Purification of CTB

For plant-produced CTB, clarified leaf extracts were adjusted to pH 8.0 with a Tris, pH 9.0 buffer. Leaf extracts and *E. coli* culture supernatants were filtered (0.22 µm) and CTB proteins were purified using the following procedure. Chromatography was performed using an AKTA Purifier (GE Healthcare). Talon Superflow Metal Affinity Resin (Clontech), packed in an XK-26 column (GE Healthcare) to a 50 ml bed volume, was equilibrated with 10 column volumes (CV) of buffer A (20 mM Tris-Cl, pH 8.0, 500 mM NaCl). Samples were loaded at 2.5 ml/min. The column was washed with 8 CV of buffer A. The CTB was eluted with a step gradient using 100% buffer B (Buffer A+150 mM imidazole) and collected by monitoring absorbance at 280 nm. SDS-PAGE was employed to assess the collected fractions for the presence of CTB. The CTB-containing fractions were further purified using a Bio-Rad CHT Hydroxyapatite Fast Flow 5 ml pre-packed column. The column was equilibrated with 10 CV of CHT buffer A (10 mM Tris-Cl, pH 8.0, 5 mM sodium phosphate) and the samples were loaded at a flow rate of 2.5 ml/min followed by a 10 CV wash with buffer A. Proteins were eluted using a gradient from 0 to 100% CHT buffer B (10 mM Tris-Cl, pH 8.0, 500 mM sodium phosphate) over 20 CV. Five ml fractions were collected and the CTB-containing fractions, after verification by SDS-PAGE, were combined, and endotoxin contaminants were removed using a Triton X-114 phase separation method [42]. Then, CTB was ultrafiltrated and diafiltrated into sterile Dulbecco's PBS (DPBS) (Gibco) using Amicon Ultra-15 3000 MWCO centrifugal devices (Millipore) according to the manufacturer's instructions. Endotoxin levels were checked with a Charles River PTS Endotoxin test system (Charles River). The concentrations of purified e- and pCTB were determined using theoretical extinction coefficients at 280 nm of 0.8181 (mg/ml)^−1^ cm^−1^ and 0.7660 (mg/ml)^−1^ cm^−1^, respectively. The molecular mass of purified pCTB was analyzed by matrix-assisted laser desorption/ionization time-of-flight (MALDI-TOF)-MS at Columbia University Medical Center Protein Core Facility. Under the conditions used, there is a matrix artifact at +202 Da next to the MH+ peak. An internal standard of myoglobin was added as an internal calibrant.

### SF-HPLC

The chromatography was performed on a Beckman Coulter System Gold HPLC. An aliquot (17 µl) of pCTB at 1 mg/ml was applied, at 1.0 ml/min, to an SEC column (YMC-Pack Diol-200, 500×8.0 mm I.D., S – 5 µm, 20 nm) equilibrated with 100 mM sodium phosphate, pH 7.0, 200 mM NaCl. After injection, 100 mM sodium phosphate, pH 7.0, 200 mM NaCl was applied to the column at a flow rate of 1.0 ml/min for 35 min. Before and after analysis, an aliquot (17 µl) of Gel Filtration Standard (Bio-rad) was applied to the column to confirm the integrity of the SEC results. CTB elution was monitored by absorbance at 280 nm.

### Biochemical analysis

Competitive GM1-ELISA and hemagglutination assay were performed as described previously [25], [30], [40], [41] except for the competitive ELISA; HRP-CTB (Molecular Probes), at the concentration of 2 µg/ml, was used as a competitor. Fifty percent inhibitory concentrations (IC_50_) were determined by the GraphPad Prism 5.0 (GraphPad Software). The binding affinity (*K*_d_) of GM1 ganglioside to CTB was measured using a Biacore ×100 2.0 instrument at ambient temperature. Briefly, mouse monoclonal antibodies to β subunit Cholera Toxin (Abcam) (mCTB; 25 µg/ml), were immobilized on a CM5 sensor chip (Biacore) in 10 mM sodium acetate pH 5.0 with a flow rate of 5 µl/min and a 10,000 resonance units (RU) target. A reference flow cell was immobilized with mCTB to correct response contributions such as bulk shifts that occur equally in the sample and reference flow cells. CTB was captured on the mCTB chip with a 200 RU target. Serial dilutions of GM1-ganglioside were made in running buffer (HPS-EP, GE Healthcare) and injected, at a flow rate of 5 µl/min, for a contact time of 60 s and a dissociation time of 600 s. A blank cycle (running buffer) was performed and all sample injections were blank subtracted to correct the sensorgrams for drifts and other disturbances that affect the reference subtracted curve. Between sample injections the system was washed with running buffer and the immobilized surface was regenerated with 10 mM glycine-HCl pH 2.5, 0.05% p20 (10% aqueous solution of polysorbate 20, GE Healthcare) for a contact time of 30 s. A replicate of a non-zero concentration of GM1-ganglioside and the blank were injected in each experiment for double referencing thus verifying the reliability of immobilized chip throughout the experiment. The data were analyzed using the 1∶1 binding kinetics analysis in the Biacore ×100 2.0 evaluation software.

### Fluorescence-based thermal shift assay (TSA)

The melting temperatures (*T*_m_) of e- and pCTB were determined by the TSA performed on a Bio-Rad iQ5 multicolor real-time PCR system. Each CTB sample, at a final concentration of 25 µM in PBS, was mixed with a final concentration of 5×Sypro orange (Molecular Probes #S-6650) for a total volume of 20 µl/well in a 96 well plate (USA scientific). Blank controls were set up for protein alone. Samples and blanks were analyzed in triplicate. The plate was heated from 20°C to 95°C in 0.2°C increments at intervals of 15 s. Data were plotted using the GraphPad software. For the acid stability, both, e- and pCTB were diluted to 1.0×10^−4^ M in the appropriate buffer and pH was checked in each sample. The buffers consisted of 100 mM sodium citrate (pH 3.0), 150 mM sodium chloride; 100 mM sodium citrate (pH 4.0), 150 mM sodium chloride; 100 mM sodium citrate (pH 5.0), 150 mM sodium chloride; 100 mM sodium phosphate (pH 6.0), and 150 mM sodium chloride; PBS (pH 7.4).

### Immunofluorescence and flow cytometry

For immunofluorescence studies, mouse monocyte Raw 264.7 (American Type Culture Collection [ATCC]) cells were cultured in chamber slides at 100,000 cells/cm^2^ in the presence of 10 µg/ml eCTB, pCTB, commercial CTB (Sigma Aldrich), or DPBS for 48 h in a humidified environment with 5% CO_2_ at 37°C. Analyses were performed in duplicates. After removal of cell culture supernatants, cells were washed and fixed with 1% paraformaldehyde (PFA) in DPBS plus Ca^2+^ and Mg^2+^ (DPBSA). Cells were permeablized with 0.5% tween-20 in DPBSA for 10 min at RT. After 2 h blocking at RT in 5% normal rabbit serum (Jackson ImmunoResearch), the cells were incubated in goat anti-CTB (List Biological) for 2 hours at RT. Finally, cells were washed and incubated with Rabbit anti-goat IgG Cy3 (Jackson ImmunoResearch) for 1 h at RT in the dark; followed by a 5 min staining with 1 µg/ml 4′,6′-diamidino-2-phenylindole (DAPI; Molecular Probes) in the dark. Images were acquired at 40× magnification on a Zeiss Observer.Z1 and processed with Axiovision AxioVs40 V4.6.30 software. Flow cytometry was used to assess the binding of e- and pCTB molecules to RAW264.7, according to a well-established procedure [43]. Briefly, 5×10^5^ cells were seeded in a 24 well plate in the presence of 10 µg/ml e- or pCTB and incubated for 24 h in a humidified environment with 5% CO_2_ at 37°C. Next, cells were washed and blocked with purified rat anti-mouse CD16/CD32 (Mouse BD Fc Block; BD Biosciences) on ice. Then cells were exposed to goat anti-CTB antibodies (List Biological Laboratories) for 1 h on ice and washed before incubation with Cy3-conjugated AffiniPure rabbit anti-goat IgG (Jackson ImmunoResearch) for 20 min on ice. Finally, cells were washed and analyzed with a FACSCalibur (Becton Dickinson), counting 10,000 cells per sample. Data were acquired and analyzed using CellQuest Pro from BD and PBS was used as a negative control.

### Animal housing

Mice were acclimated for approximately one week prior to the initiation of the studies. 8 week-old female C57BL/6J mice were purchased from The Jackson Laboratory in Bar Harbor (Maine, USA). Four animals were housed in each filtertop microisolator cage in a temperature- and humidity-controlled room with alternating light/dark cycles of 12 h, with access to Laboratory Autoclavable Rodent Diet 5010 (LabDiet) and water *ad libitum*.

### Mouse immunization and sample collections

Mice were randomly divided into groups of 4, and eCTB or pCTB (10 or 30 µg), or PBS vehicle control in a volume of 100 µl was administered orally on days 1 and 15 after neutralization of stomach acids using 200 µl of 30 mg/ml sodium bicarbonate solution. No adjuvant was used for immunization. Blood samples were collected from the submandibular vein in BD Microtainer serum separator tubes (Becton Dickinson) and spun at 6000×g for 5 min to obtain the serum samples. Dry fecal pellets were collected at multiple time points during the experiment as indicated in the results. Fecal samples were prepared as described previously [44] with some modifications. Typically, 100 mg of fecal pellets were homogenized in 1 ml cold 0.05% sodium azide in PBS and the homogenates clarified twice by centrifugation at 10,000×g at 4°C for 10 min. In the Chinese hamster ovary (CHO) cell elongation assay, fecal extract preparation buffers did not include sodium azide and the samples were precipitated with 80% ammonium sulfate, dialyzed against PBS, and filtered through a 0.45 µm Supor membrane (Life Sciences).

### Quantification of mucosal and systemic Igs

Total and anti-CTB specific IgA and IgG levels were determined in fecal extracts and serum samples, respectively, using ELISA as previously described [25], [44]. Briefly, MaxiSorp ELISA plates (Nalgene Nunc International) were coated overnight with 1 µg/ml purified eCTB. After blocking, 50 µl of fecal or serum samples in appropriate dilutions were added to the plates. Fecal IgA bound to CTB were detected using goat anti-mouse IgA antibodies conjugated with HRP (SouthernBiotech) and a SureBlue TMB 1-Component Microwell Peroxidase Substrate (KPL, Gaithersburg). Absorbance was measured at 450 nm with a background reading at 570 nm in a Synergy HT plate reader (BioTek) after the reaction was stopped. Serum IgG were quantified similarly using HRP-conjugated anti-mouse IgG antibodies (SouthernBiotech). Dilutions of purified mouse IgA or IgG standards were used to calibrate the ELISA. The titer was defined as the greatest dilution factor of the sample with positive OD_450_ reading after subtracting an average background value.

### Lamina propria lymphocyte (LPL) isolation

Lymphocytes from mouse intestinal lamina propria were isolated according to a well-established protocol which involves the removal of Peyer's patches and a series of collagenase digestions of tissue to produce a single-cell suspension [45]. LPLs from the same group of animals were pooled for subsequent analysis.

### B-cell enzyme-linked immunospot (ELISPOT) assay

B-cell ELISPOT method was carried out using standard procedures [46], [47]. Briefly, MultiScreen-Filter plates (Millipore) were coated with e- or pCTB (antigen specific) in PBS and blocked with complete RPMI medium at room temperature. After washing, dilutions starting at 10^7^cells/ml LPLs were plated and incubated for 4 h at 37°C in a humid environment with 5% CO_2_. Plates were then washed thoroughly and incubated with goat anti-mouse IgA HRP conjugate (SouthernBiotech) overnight at 4°C. Finally, the plates were washed and developed at RT for 10–60 min using freshly prepared amino ethyl carbazole (AEC) substrate solution and the reaction was stopped with distilled water. Spots were counted in each well, using an ImmunoSpot Reader (Cellular Technology, Ltd.) of air dried plates and results expressed as the average value of triplicate wells normalized to the counted number of spots per 10^6^ cells after background correction. For total spot count, the plates were submitted to the same procedure with the notable exception that the wells were coated with rat anti-mouse IgA instead of CTB.

### Functional analyses of anti-CTB antibodies

The ability of mouse anti-CTB antibodies to bind CT holotoxin was assessed in GM1-ELISA [44]. MaxiSorp ELISA plates were coated overnight with 2 µg/ml monosialogangliosides GM1 (Sigma-Aldrich) at 4°C. Samples consisting of 100 ng/ml CT were pre-incubated for 1 h at 37°C with equal volumes of protease free fecal extracts or 10-times-diluted serum samples from immunized mice. Fifty µl of each sample was added to the plates followed by 1 h incubation at 37°C. Following a wash step, the plates were incubated with primary anti-CT antibodies (Sigma-Aldrich) for 1 h at 37°C. HRP-conjugated goat anti-rabbit secondary antibodies and the TMB substrate were used for detection as described for antibody titer analysis. The results were expressed as percentages of an average OD value signal obtained from samples treated with sera or fecal samples from PBS immunized animals.

### CHO cell elongation assay

CHO AA8 cells [48], [49] from ATCC were cultured according to the manufacturer's instructions. Cell elongation assays were carried out as previously described [50], with some modifications. In brief 2,500 CHO AA8 were seeded per well in a 24-well cluster overnight. Ten times diluted fecal or serum samples were added to equal volumes of CT at a final concentration of 10 ng/ml and incubated in a water bath at 37°C for 1 h. Next, the cells were washed and 50 µl of the pre-incubated samples (Antibodies/CT) were added to 450 µl culture medium and used to treat CHO cells overnight. After staining the cells with Crystal Violet, cell length was measured using a Nikon Eclipse TE300 Microscope and the Metamorph software.

### Statistical analyses

Group means, standard deviations (SD), and standard errors of the mean (SEM) were derived from the values obtained in at least 3 independent replicates. Statistical significance was analyzed by a one-way analysis of variance (ANOVA) with Bonferroni's multiple comparison test or student's *t*-test unless otherwise stated, using the GraphPad Prism 5.0 software. Differences were considered statistically significant if *P*<0.05.

## Results

### Recombinant CTB expressed in transgenic *N. benthamiana* is *N*-glycosylated

Upon obtaining kanamycin-resistant transformants, CTB-expressing lines were screened by GM1-ELISA. The CTB expression level of the selected line was up to 0.5 mg of the protein per kg of leaf material (data not shown).

**Figure 1:**
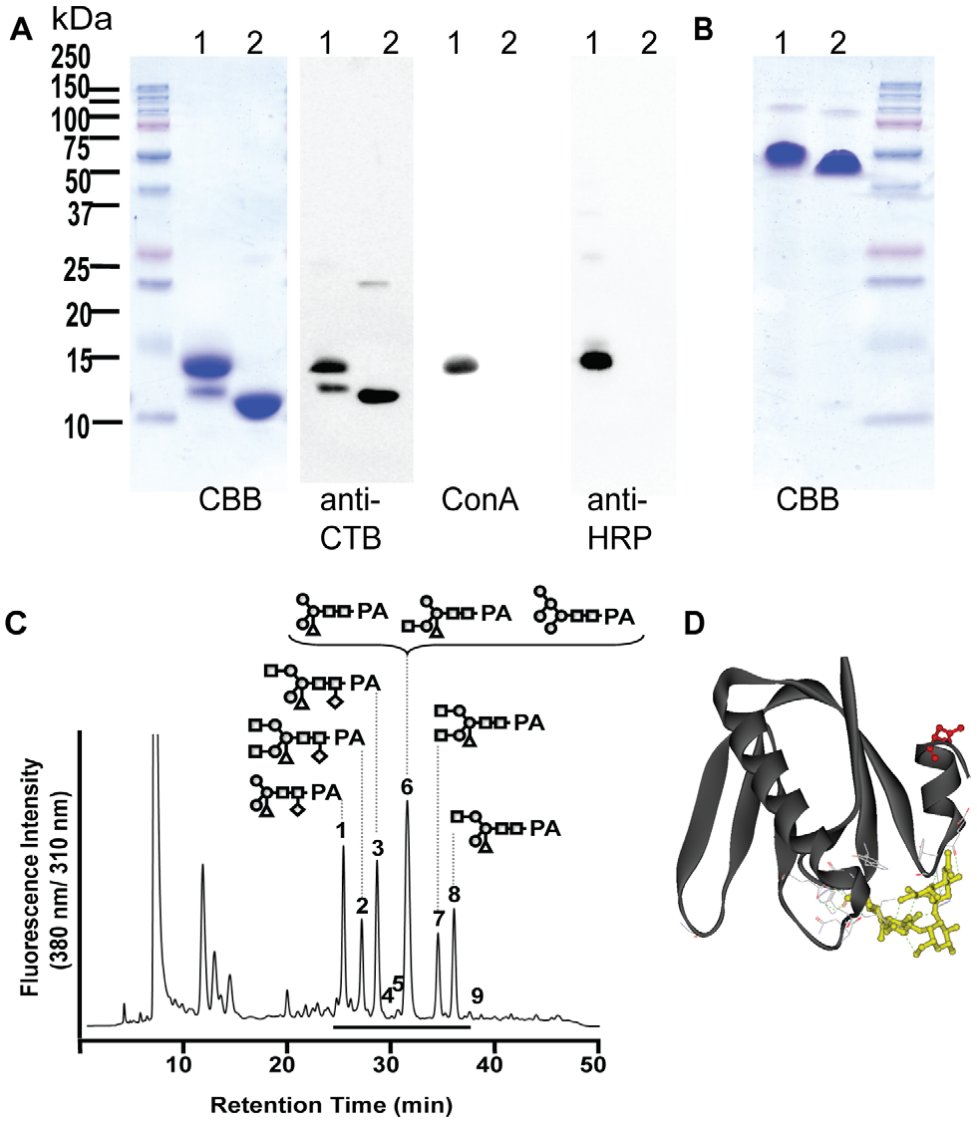
Transgenic *N. benthamiana*-expressed CTB is *N*-glycosylated. *A*, denaturing SDS-PAGE (Coomassie Brilliant Blue Stained, CBB) and an anti-CTB immunoblot showed that transgenic plant-expressed CTB has two monomer species, whereas the *E. coli*-produced counterpart has only one. The lectin concanavalin A (ConA) and anti-peroxidase antibodies (Anti-HRP) both bound to the upper band of the plant-expressed CTB, but not to the lower band or the eCTB, demonstrating the presence (in the upper band) and absence (lower band) of plant *N*-glycans in the plant-expressed B subunit (Lane 1 – CTB purified from transgenic *Nicotiana benthamiana*; 2 – eCTB) *B*, non-heat denaturing SDS-PAGE showed that transgenic *Nicotiana*-expressed CTB and the *E. coli-*made counterpart were assembled into homo-pentamers. The lane numbers are the same as in Fig. 1*A*. *C*, analysis of *N*-glycan structure showing the presence of plant-specific α(1, 3)-fucose/β(1,2)-xylose-containing glycans. The chromatogram shows RP-HPLC separation of 2-aminopyridine (PA)-labeled glycans isolated from *Nicotiana*-expressed CTB, with representative glycan structures depicted at corresponding PA-glycan fractions. A horizontal line represents the elution position for PA-labeled glycans. *Symbols*: circle (○), mannose; square (□), N-acetylglucosamine; diamond (◊), fucose; and triangle (Δ), xylose. *D*, a crystal structure image showing a CTB monomer complexed with GM1-ganglioside (side view; PBD ID: 3CHB). Dot lines in green represent hydrogen bonds involved in binding to GM1-ganglioside, which is shown in yellow. Asn4, which is glycosylated upon expression in eukaryotic cells, is highlighted in red. It is evident that the Asn4 side chain does not interact with GM1-ganglioside. Image was created in Accelrys Discovery Studio Visualizer 2.5.

To characterize the transgenic *Nicotiana*-expressed recombinant CTB in detail, we purified the protein from leaves using immobilized metal affinity chromatography (IMAC) by taking advantage of CTB's natural chelating activity [51], followed by an additional chromatography step using a hydroxyapatite resin. SDS-PAGE analysis of the purified protein under heat-denaturing conditions revealed two distinct bands; a major band at ∼14.5 kDa and a minor one at ∼12.5 kDa. Non-heat-denaturing conditions showed a single band at 60–70 kDa (Fig. 1A, B). The molecular size of the mature CTB monomer deduced from its primary sequence was 12.3 kDa, corresponding well to the size of the lower band under the heat-denatured conditions. Immunoblot analysis using anti-CTB Abs showed that both bands were indeed CTB-related proteins (Fig. 1A). eCTB (devoid of the C-terminal SEKDEL sequence, with a predicted molecular size of 11.6 kDa) migrated slightly faster than the lower band of plant-expressed protein, suggesting that partial degradation did not give rise to the two sub-species of plant-expressed CTB. Based on previous findings on the possible *N*-glycosylation of CTB, we postulated that the heterogeneity might be attributed to the inefficient recognition of nascent CTB polypeptides by the oligosaccharyltransferase complex in the ER [31], [32]. Accordingly, lectin- and immuno-blot analysis using ConA and anti-peroxidase Abs (the latter recognizes Xyl- and Fuc-containing plant glycans; for example, see [52]) were performed. The results clearly showed that the upper band, but not the lower one, is modified with plant *N*-glycans (Fig. 1A). The cause of such inconsistent glycosylation is unclear, but not uncommon for recombinantly produced glycoproteins [53], [54].

To further dissect the *N*-glycosylation of *Nicotiana*-expressed CTB, we analyzed its glycan composition and structure using a combination of comparative HPLC and MS. Nine distinctive fractions of PA derivatives were identified (Fractions 1–9; Fig. 1C), each of which was subsequently subjected to SF-HPLC to separate further (Fig. S1). Table 1 depicts the composition of *N*-glycans attached to transgenic *Nicotiana*-expressed CTB. In spite of the ER retention signal attached to the C-terminus of CTB, which would theoretically provide mannose-rich Man5-9GlcNAc2 oligosaccharides, the majority (>80%) showed more processed glycoforms containing a plant-specific β(1,2)-linked xylose and/or an α(1,3)-linked fucose.

**Table 1:** *N*-glycan composition of CTB expressed in transgenic *N. benthamiana*.

Structure^a^		HPLC fraction^b^	Relative amount (%)^c^	
Oligomannosidic glycans	Man_9_GlcNAc_2_	2-j	0.4	16.0
	Man_8_GlcNAc_2_	1-i	2.1	
	Man_7_GlcNAc_2_ d	1-h/3-h	0.4/0.7	
	Man_6_GlcNAc_2_	4-d	3.1	
	Man_5_GlcNAc_2_	6-d	9.3	
α(1,3)-fucose/β(1,2)-xylose-containing glycans	Man_3_Xyl_1_GlcNAc_2_	6-b	16.5	83.3
	Man_3_Fuc_1_GlcNAc_2_	1-b	1.2	
	Man_3_Xyl_1_Fuc_1_GlcNAc_2_	1-d	15.0	
	GlcNAc_1_Man_3_Xyl_1_GlcNAc_2_ d	6-c/8-c	6.0/11.9	
	GlcNAc_1_Man_3_Xyl_1_Fuc_1_GlcNAc_2_ d	1-f/3-f	4.0/12.3	
	GlcNAc_2_Man_3_Xyl_1_GlcNAc_2_	7-c	8.4	
	GlcNAc_2_Man_3_Xyl_1_Fuc_1_GlcNAc_2_	2-g	7.9	
Other glycans	GlcNAc_1_Man_3_GlcNAc_2_	9-b	0.3	0.7
	GlcNAc_2_Man_3_GlcNAc_2_	7-b	0.4	

a Man, mannose; GlcNAc, N-acetylglucosamine; Xyl, xylose, and Fuc, fucose.

b Fraction numbers are shown in Fig. 1C and Fig. S1.

c The relative amount of each glycan was calculated from the fluorescence intensity of PA fractions in HPLC.

d Two isomers for the terminal GlcNAc residue were identified.

### High-level expression of a non-glycosylated CTB variant, pCTB, pentamer was obtained by virus vector-based expression

To eliminate *N*-glycosylation, we performed site-directed mutagenesis to mutate the AAC codon (Asn4) (amino acid numbering based amino acids 22–126 of GenBank accession no. AY475128 without the secretory signal)→AGC (Ser). Serine was chosen because the closely related *E. coli* heat-labile enterotoxin B subunit has Ser at the corresponding position (for example, see GenBank accession no. AAC60441), and when examining the structure of CTB, Asn4 is not involved in GM1 binding nor does the Asn side chain stabilize the homo-pentamer structure (Fig. 1D). Thus, we anticipated that such a mutation will not affect CTB's structure or function.

**Figure 2:**
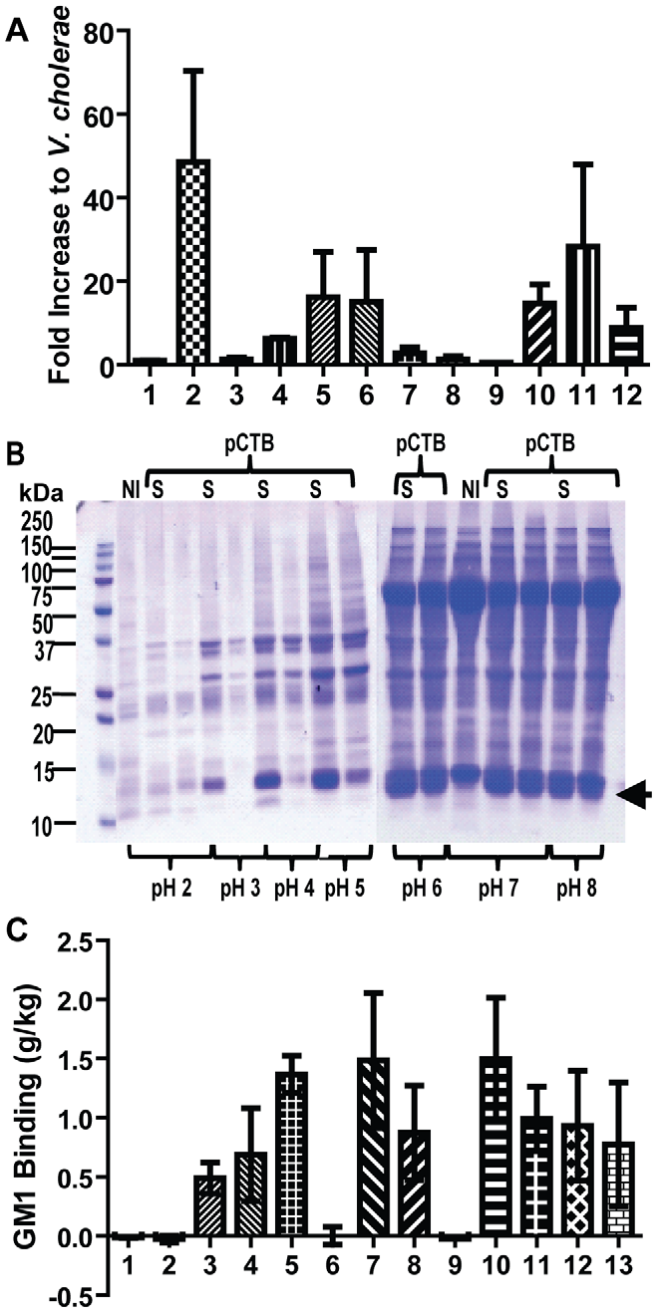
Tobamoviral vector-based overexpression of the aglycosylated variant pCTB in *N. benthamiana*. *A*, quantification of pCTB accumulation in leaf extracts with twelve different secretory signals. Lane 1- *V. cholerae* CTB; 2 - Rice-α amylase; 3 - *N. plumbagenifolia* calreticulin; 4 - Apple pectinase; 5 - Barley-α amylase; 6 - *H. Vulgare* chitinase; 7 - *S. Tuberosum* Glucan endo-1,3-beta-D-glucosidase; 8 - *A. Thaliana* Auxin-binding protein 1; 9 - *P. Atrosepticum* Pel B; 10 - *P. vulgaris* endopolygalacturonase-inhibiting protein; 11 - Tobacco PR1a; and 12 - Rice glutelin. GM1-ELISA was employed to quantify the amounts of receptor-binding pCTB. Data are expressed as mean ± SEM of experimental triplicate. The levels were normalized to an average accumulation level obtained with *V. cholerae* CTB signal peptide (0.016 g/kg). A representation of two separate experiments is shown. *B*, SDS-PAGE analysis of crude extracts of *N. benthamiana* leaves expressing pCTB with rice-α amylase signal peptide. Five days post vector inoculation (dpi), leaf proteins expressing pCTB were extracted with different pH buffers without or with 0.5 M sodium chloride salt (S). The arrow indicates the position of pCTB at ∼12 kDa. Control leaf extract are labeled NI. Extraction at pH 5 effectively removed ribulose-1,5-bisphosphate carboxylase oxygenase for simple and efficient pCTB purification based on chromatography. *C*, quantification of pCTB expressed with rice-α amylase signal peptide in leaf extracts (5 dpi). Data, expressed in gram of pCTB per kg of leaf material, were obtained by GM1-ELISA and plotted as mean ± SEM of three separate production runs. Column numbers 1–12 correspond to NI pH 2 with salt, pCTB pH 2 with salt, pCTB pH 3 with salt, pCTB pH 4 with salt, pCTB pH 5 with salt, pCTB pH 5, pCTB pH 6 with salt, pCTB pH 6, NI pH 7 with salt, pCTB pH 7 with salt, pCTB pH 7, pCTB pH 8 with salt, pCTB pH 8, respectively.

**Figure 3:**
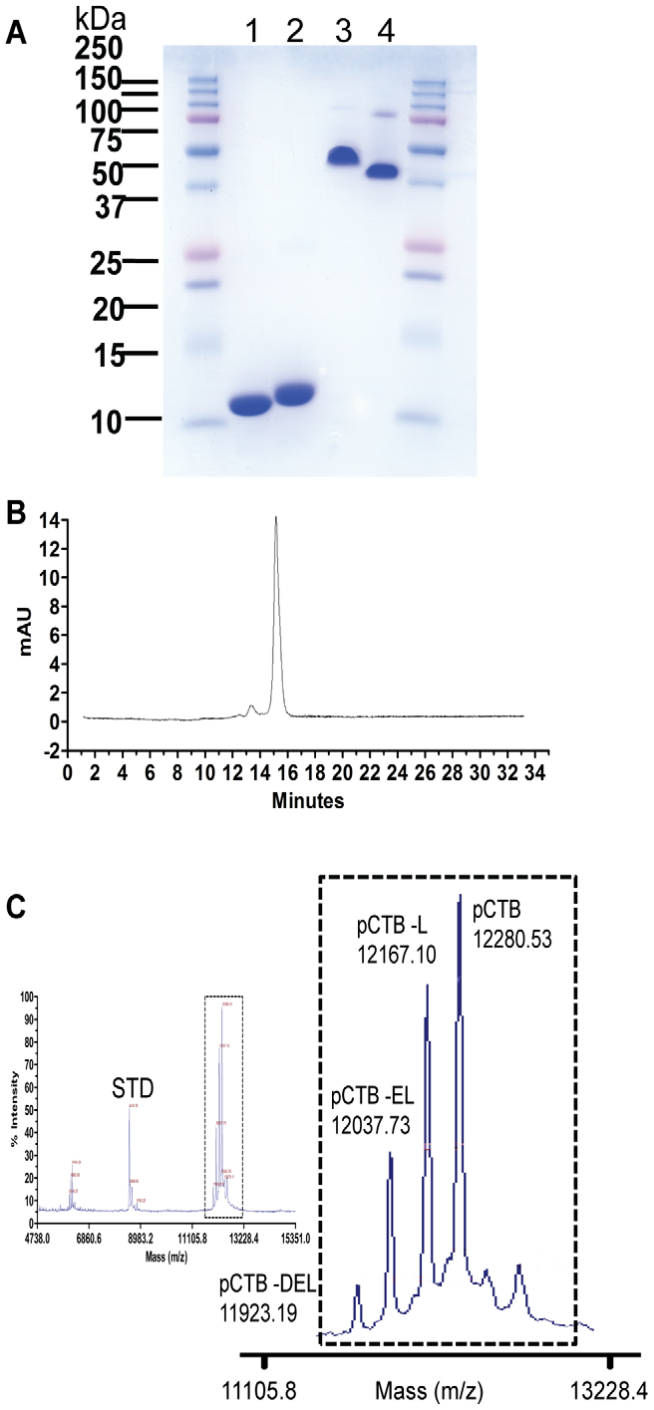
Purity of pCTB. *A*, SDS-PAGE analysis. eCTB (lanes 1 and 3) and pCTB (lane 2 and 4) were purified by IMAC and a hydroxyapatite resin as described in Experimental Procedures. A total of 10 µg of purified protein was loaded in each well. Both CTB proteins appear as monomers under heat denaturing conditions (lanes 1 and 2), whereas mostly as pentamers with a trace amount of decamers under non-heat-denaturing conditions (lanes 3 and 4). *B*, SF-HPLC analysis of pCTB. The large peak corresponds to the CTB pentamer purified to >95% homogeneity. The minor peak is likely a decamer form (see text for details). *C*, MALDI-TOF-MS analysis of pCTB. The ions (peaks) in the spectrum of singly charged species ([*M*+H]^+^; magnified in the right panel) contain the mass-to-charge-ratios (*m/z*) of 12280.53, 12167.10, 12037.73, and 11923.19. These correspond well to the theoretical *m/z* values of full-length pCTB (12281.98), and variants lacking the C-terminal Leu (12168.78), Glu-Leu (12039.66), and Asp-Glu-Leu (11924.58), respectively.

Making stable transgenic lines is time consuming and generally yields low accumulation of foreign proteins (∼1% of total soluble proteins) [42]. Hence, we chose a deconstructed tobamovirus-based vector system [55], [56] to overexpress various pCTB variants in *N. benthamiana*. GM1-ELISA on crude leaf extracts revealed that the aglycosylated B subunit retained a pentameric form although expression was low, i.e., less than 0.1 g/kg (Fig. 2A, column 1). We hypothesized that changing the secretory signal may lead to an increase in pCTB accumulation. Thus, the original *V. cholerae* secretory signal (corresponding to amino acids 1 to 21; GenBank accession no. AY475128) was replaced with various other signals of plant origin. GM1-ELISA showed that a secretory signal derived from rice-α amylase provided the highest accumulation of pCTB at 0.5–1.5 g/kg of leaf material (Fig. 2A and C), representing ∼40-fold increase from the *V. cholerae* secretory signal construct. An extraction profile showed that soluble pCTB pentamers were extracted over a wide pH range, pH 3–8. (Fig. 2B). The pH 5 with salt extract was chosen given that the condition provided the best yields of GM1-binding CTB (Fig. 2C) while removing majority of host-derived proteins including ribulose-1,5-bisphosphate carboxylase oxygenase (Fig. 2B), which will aide in purification. High expression is not unusual for the vector used here; however, our expression level is among the highest for plant-based recombinant protein expression reported to date [42]. Importantly, pCTB is not *N*-glycosylated, as evident from a single band at 12.5 kDa corresponding to the CTB subunit monomer without a glycan (Fig. 2B, 3A), MS analysis (see below), and ConA blot (data not shown). Collectively, these results indicated that *N*-glycosylation is not necessary for the overexpression of GM1-ganglioside-binding CTB pentamers.

### pCTB retains nanomolar GM1-ganglioside binding affinity and physicochemical stability

The observation that pentameric pCTB was expressed at a remarkably high level prompted us to purify the plant-derived aglycosylated B subunit for additional feasibility assessment. Specifically, it was essential to demonstrate that pCTB is comparable to the native bacterial counterpart as the former has Asn4→Ser mutation and a C-terminal ER retention signal. Extraction with a simple aqueous buffer at pH 5 (Fig. 2B and C) followed by two-step chromatography based on IMAC and hydroxyapatite yielded a highly purified protein, as demonstrated by SDS-PAGE analysis and SF-HPLC. The denaturing SDS-PAGE (Fig. 3A) showed each CTB in its monomeric form whereas the non-heat-denaturing SDS-PAGE (Fig. 3A) illustrated that both CTBs retain pentamer formation. Both, e- and pCTB had a single band and showed >95% purity (based on a densitometric analysis). On the SF-HPLC chromatogram (Fig. 3B), pCTB exhibited one large peak and a small peak at a shorter retention time. Because CTB subunits can form a decameric structure [20], the small peak may represent the decameric form of pCTB. The elution time of the large peak roughly corresponded to the size of a pentameric form (50–60 kDa) and therefore the purity of the pentameric form with trace amounts of the decamer was estimated to be >95% for pCTB. MALDI-TOF-MS (Fig. 3C) was performed to determine the actual mass of pCTB. The predicted molecular mass of pCTB was 12281.98 Da. The spectrogram demonstrated that pCTB had a defined peak at 12280.53 Da, thus corresponding well to the expected molecular mass (<0.02% deviation). The approximately 2 Da difference was likely caused by a loss of 2 hydrogen atoms due to the formation of a disulfide bond between the two cysteines, presumably the intramolecular disulfide bond between Cys-9 and Cys-86, which is essential for proper protein folding and GM1-binding activity [7], [57], [58]. The peaks at 12167.10, 12037.73, and 11923.71 Da corresponded to pCTB variants lacking the C-terminal Leu, Glu-Leu, and Asp-Glu-Leu, respectively. The truncation of the ER retention signal did not affect the functionality of the protein (see below).

**Figure 4:**
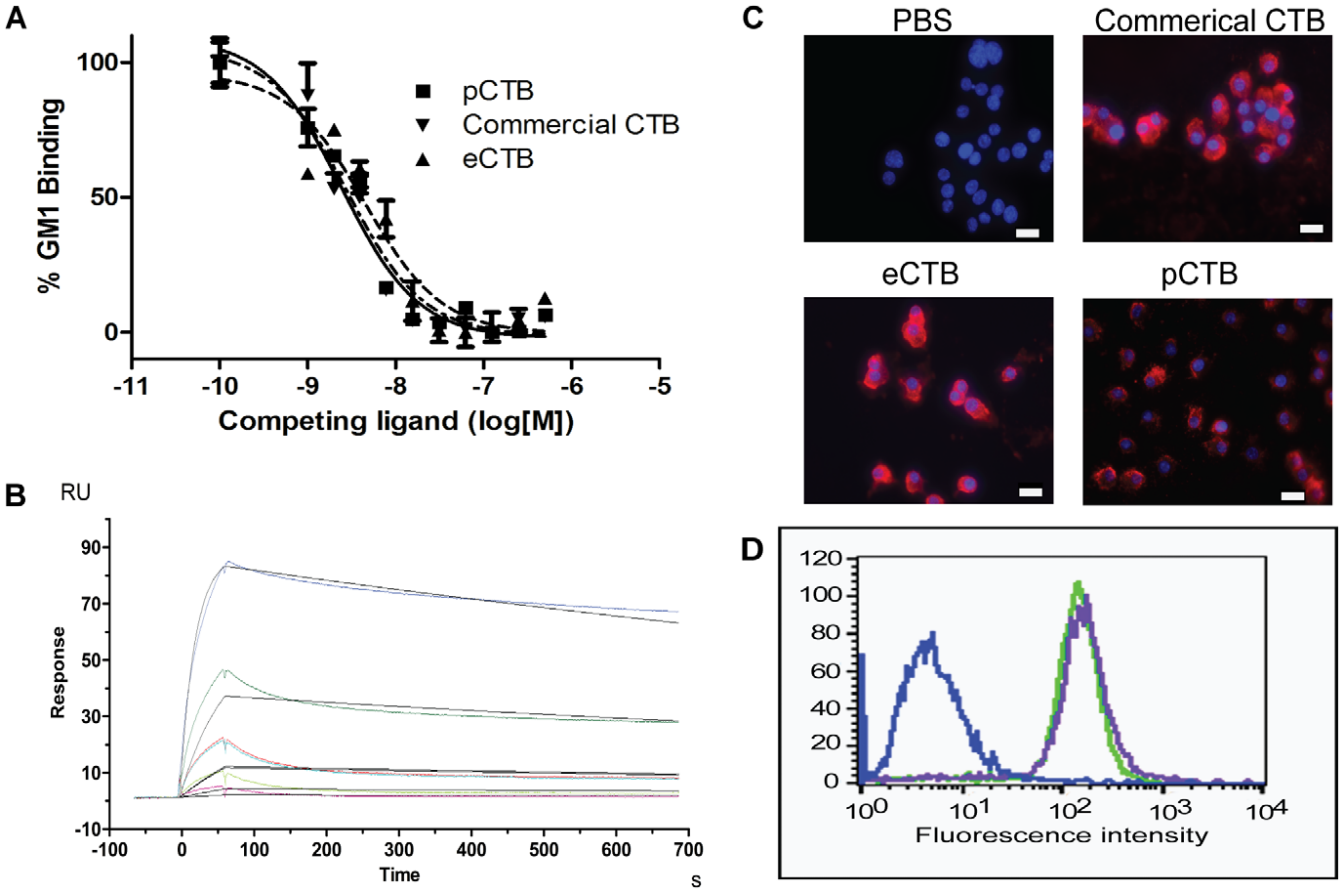
Characterization of pCTB's GM1-ganglioside-binding activity. *A*, binding affinity determination based on competitive GM1-ELISA using HRP-labeled CTB. The assay was performed in triplicate. The 50% inhibitory concentrations (IC_50_) of commercial, p-, and eCTB were determined by non-linear regression analysis (GraphPad Prism 5.0) to be 2.44, 2.85, and 4.51 nM, respectively. *B*, SPR. Each CTB protein was immobilized on a sensor chip via a CTB-specific monoclonal antibody, and varying concentrations of GM1-ganglioside were used as analytes. For each protein the assay was performed in triplicate. A representative sensorgram obtained with pCTB is shown. The colored curves represent the concentration of GM1-ganglioside (10, 3.33, 1.11, 0.37, and 0.123 µg/ml from top to bottom), and the black lines are the 1∶1 binding kinetics fit. *C*, analysis of pCTB's binding to RAW264.7 by immunofluorescence. After 48 h incubation, binding of (a) a PBS control, (b) commercial CTB, (c) pCTB, and (d) eCTB to the cells was detected by anti-CTB primary and Cy3-conjugated secondary antibodies on a Zeiss Observer.Z1 microscope (40×magnification) with the Axiovision AxioVs40 V4.6.30 software. The red signals correspond to the presence of CTB proteins. Nuclei were counterstained with DAPI. No significant difference was noted in cell-surface binding or cell morphology among the three proteins. *Scale bar*, 20 µm. *D*, a representative flow cytogram showing RAW264.7 cells bound by e- and pCTB, as detected by Cy3-conjugated secondary antibodies. PBS, eCTB, and pCTB histograms are blue, purple, and green, respectively, demonstrating the comparable binding of the bacterial and plant-made aglycosylated B subunits to the cells.

To test whether pCTB retains high affinity for GM1-ganglioside, a competitive GM1-ELISA was performed (Fig. 4A). There was no significant difference among the apparent affinities of commercial and the two recombinant CTBs to the receptor; commercial CTB (Sigma-Aldrich), pCTB, and eCTB showed 50% inhibitory concentrations (IC_50_) of 2.44, 2.85 and 4.51 nM, respectively. A haemagglutination assay with GM1-ganglioside-coated red blood cells [41] also revealed that both p- and eCTB displayed a similar haemagglutination effect in nanomolar range (Fig. S3). Lastly, surface plasmon resonance was performed to determine the dissociation constant, *K*_d_ (Fig. 4B). Employing the Biacore ×100 1∶1 binding kinetic analysis, it was found that commercial CTB, pCTB, and eCTB had *K*_d_ values of 51.40±5.69, 52.58±5.68, and 53.20±1.25 nM, respectively. There was no statistically significant difference between the three *K*_d_ values.

We also assessed pCTB's capacity to bind to cell-surface GM1-ganglioside. The immunocytochemistry in Fig. 4C demonstrated that commercial CTB, pCTB, and eCTB all bind to the surface of RAW 264.7 cells in a similar manner. No morphological or growth-pattern difference was noted between cells treated with the B subunits from the three different sources. Cell-surface binding was further confirmed by flow cytometry, where a clear and similar shift was observed for cells treated with e- and pCTB as opposed to control PBS treated cells which did not show any fluorescence (Fig. 4D).

**Figure 5:**
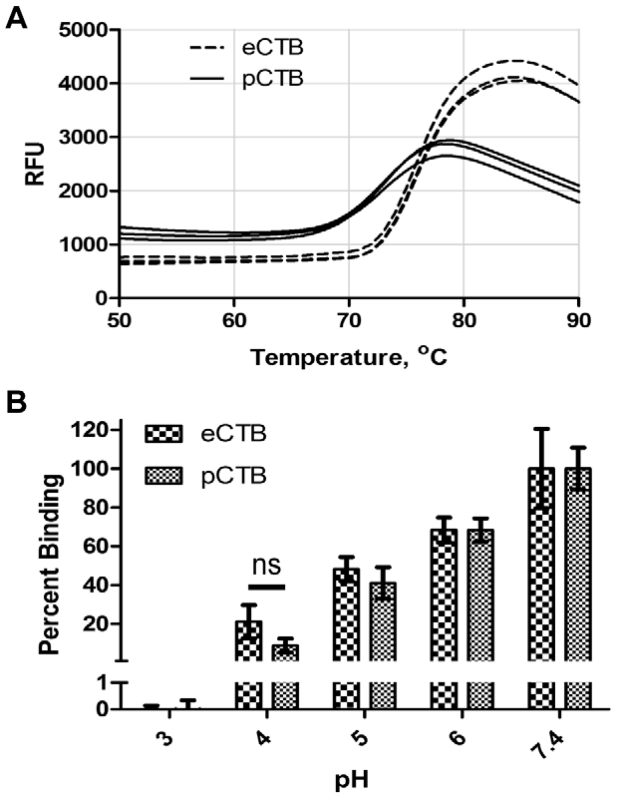
Thermal and pH stability analysis of pCTB. *A*, the melting temperature (*T*_m_) determination using TSA. eCTB (dashed line) and pCTB (solid line) were analyzed in triplicate representing three individual lines per protein in the graph. *T*_m_ of e- and pCTB were 75.5°C and 72.9°C, as determined by the vertex of the first derivative of the relative fluorescence unit (RFU) values. *B*, pH stability analysis by GM1-ELISA. e- and pCTB were diluted to 50 ng/ml in various pH buffers (see methods) and incubated for 5 min, and GM1-bound CTB proteins were quantified by GM1-ELISA. The results were normalized to the average value of the corresponding CTB protein at pH 7.4 and expressed as % binding. Data represent the mean ± SEM (n = 6) ns: values not significantly different at *P*>0.05 determined by unpaired Student *t*-test.

In order to discern potential structural instability associated with mutations introduced to create pCTB, we utilized TSA [59] to determine the *T*_m_ of the CTB variants. The bacterial and plant-produced proteins showed *T*_m_ of 75.5±0.12°C and 72.9±0.31°C, respectively (Fig. 5A) which is statistically different at *P*<0.05 as determined by the unpaired *t-*test. Albeit with the slight decrease of *T*_m_, the results indicate that the amino acid modifications placed on pCTB did not significantly affect the thermal stability of the B subunit. The acid stability of both CTBs in different pH buffers was also examined by TSA. As shown in Table 2, both e- and pCTB had similar melting temperatures at the various pH conditions studied. To corroborate these results, we performed GM1-ELISA using the proteins exposed to varying pH conditions (Fig. 5B). The results again showed that there was no significant difference in the pH stability between e- and pCTBs. Taken together, the above data demonstrated that pCTB holds physicochemical stability comparable to the original protein.

**Table 2:** pH stability of e- and pCTB determined by TSA^a^.

	*T*_m_ b	
pH	eCTB	pCTB
3.0	39.8±3.74°C	44.1±0.58°C
4.0	65.2±0.12°C	60.5±0.12°C
5.0	75.3±1.2°C	74.3±0.31°C
6.0	78.1±0.31°C	75.8±0°C
7.4	75.5±0.12°C	72.9±0.31°C

a Proteins were diluted in appropriate pH buffers (see Experimental Procedures for details).

b *T*_m_ were determined by the vertex of the first derivative of the relative fluorescence unit values. The assay was performed in triplicate and *T*_m_ are expressed as mean ± SD of triplicate.

### pCTB is orally active to induce robust antibody responses

**Figure 6:**
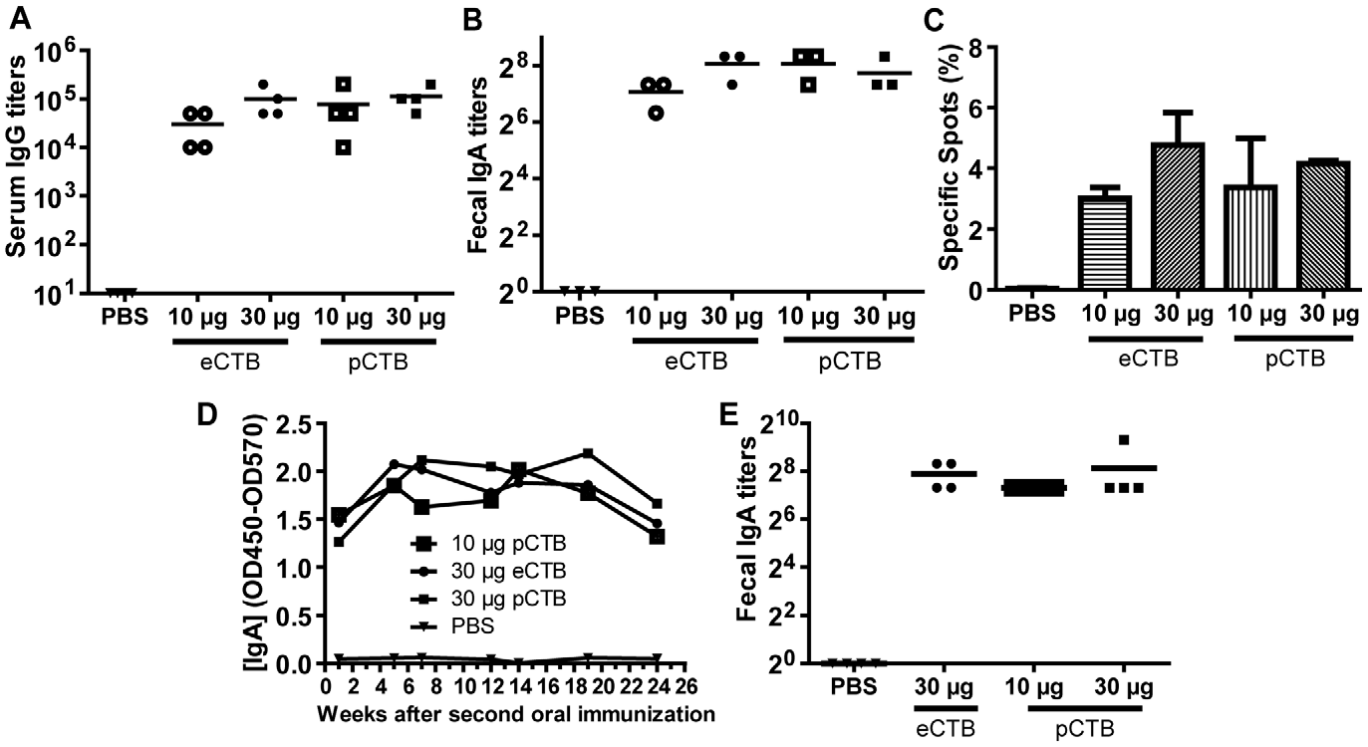
Oral immunogenicity of *N. benthamiana*-produced pCTB. *A* and *B*, anti-CTB antibody titers. C57bl/6 mice were orally immunized with PBS, eCTB, or pCTB (10 or 30 µg) and again with the same antigens 2 weeks later. Endpoint titers of serum anti-CTB IgG (*A*) and fecal anti-CTB S-IgA (*B*) were analyzed 2 weeks after the second immunization. Horizontal bars show mean titers, whereas symbols represent titers of individual mice (for serum IgG) or triplicate analysis (for fecal IgA; fecal samples of each group were pooled for analysis). There was no significant difference between titers induced by the bacterial and plant-made aglycosylated B subunits (ANOVA with Bonferroni's multiple comparison test). *C*, representation of the percentage of anti-CTB IgA antibody secreting cells (ASCs) relative to the total IgA ASCs isolated from lamina propria, as determined by B-cell ELISPOT assay. Analysis was done 2 weeks after the second immunization. Data shown are mean ± SEM of triplicate analysis. No significant difference was found among e- and pCTB-immunized groups (ANOVA with Bonferroni's multiple comparison test). *D*, time-course assessment of CTB-specific IgA levels in fecal extracts. Fecal samples were collected 2 to 24 weeks after the second immunization from mice immunized with PBS, 30 µg eCTB, or 10 or 30 µg pCTB. Anti-CTB IgA levels induced by both bacterial and plant-made aglycosylated B subunits were sustained over 6 months. *E*, fecal anti-CTB IgA endpoint titers at termination (6 months after the second immunization). Horizontal bars show mean titers. Symbols represent titers of individual mice (fecal samples were not pooled). No significant difference was found among e- and pCTB-immunized groups (ANOVA with Bonferroni's multiple comparison test).

The results shown above have demonstrated the integrity of pCTB at the molecular level. We next shifted our investigation to examining the *in vivo* activity of pCTB. Mice were orally immunized with 3, 10, or 30 µg of e- or pCTB followed by a boost 2 weeks after the initial administration. Serum samples from animals immunized with 30 µg CTB per dose showed similar anti-CTB specific IgG titers, of about 100,000 on average, for both bacterial and plant-manufactured B subunits (Fig. 6A). Serum samples from PBS treated animals did not show any reaction above the background (Fig. 6A). Likewise, fecal extracts obtained from mice immunized with 10 µg pCTB and 30 µg of either pCTB or eCTB showed comparable anti-CTB specific IgA titers above 200 (Fig. 6B). Mice treated with 10 µg eCTB showed slightly lower levels of anti-CTB specific IgA in fecal extracts compared with the other groups mentioned above although the difference was not statistically significant (Fig. 6B). Meanwhile, a weak mucosal immune reaction was obtained from the animals immunized with 3 µg of CTB regardless of the origin, plant-made or bacteria-produced, as quantified by fecal IgA and serum IgG titers (data not shown).

To dissect the CTB-induced immune response at the cellular level, the number of anti-CTB IgA secreting cells isolated from the lamina propria of small intestine were determined in an ELISPOT assay. The total spots representing all cells secreting IgA in the population was similar across groups, including PBS and CTB-immunized animals. As expected, no anti-CTB specific antibody producing cells were detected from PBS treated animals (Fig. 6C). The numbers of anti-CTB IgA-producing cells were comparable between animals that received the same dose of CTB regardless of the system used to produce the protein, whether bacterial or plant-made. There was a trend of dose dependent increase in the number of specific spots, whereby ∼4% of total IgA-secreting cells were CTB-specific with 30 µg dose. At 3 µg dose, both p- and eCTB induced ∼1% of CTB-specific IgA-secreting cells (data not shown).

We also assessed the duration of the anti-CTB specific IgA response in fecal samples after immunization. As shown in Fig. 6D, anti-CTB IgA levels remained high throughout the 6 months that the experiment lasted. At termination the animals treated with 30 µg pCTB or eCTB showed similar titers for anti-CTB specific IgA, relatively higher but not significant compared with the mice that were administered 10 µg of pCTB (Fig. 6E).

### pCTB-induced antibodies effectively neutralized cholera holotoxin (CT)

**Figure 7:**
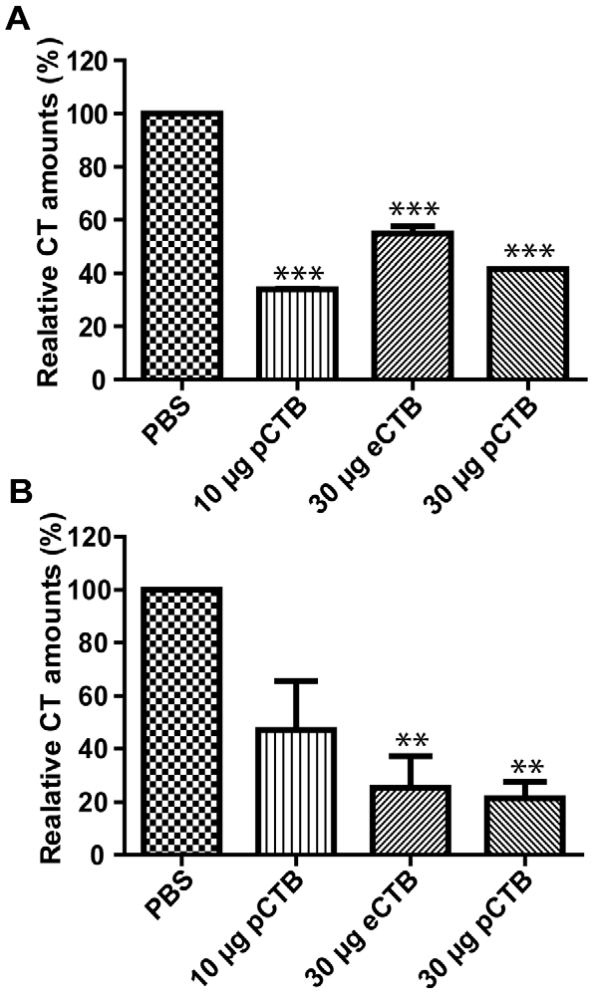
Inhibition of CT holotoxin's GM1-ganglioside binding by pCTB-induced antibodies. *A* and *B*, relative amounts of GM1-bound holotoxin in the presence of (*A*) fecal and (*B*) serum Igs. CT holotoxin (100 ng/ml) was pre-incubated with undiluted fecal extracts (*A*) and 10× diluted sera (*B*) obtained from animals orally immunized with PBS, 30 µg eCTB, or 10 or 30 µg pCTB and analyzed in a GM1-ELISA. The results are shown as % of the average amount of CT detected with the PBS control group. Data shown are mean ± SEM of triplicate. Both fecal and serum Igs were prepared from animals 2 weeks after the second vaccination. For fecal Igs, all groups showed significant inhibition. For serum Igs, groups that were immunized with 30 µg pCTB and 30 µg eCTB showed significant inhibition: **P<0.01; ***P<0.001 (ANOVA with Bonferroni's multiple comparison test).

To evaluate the efficacy of antibodies raised against p- and eCTB, we exploited CT holotoxin's ability to bind to GM1-ganglioside. As seen in Fig. 7A, incubation of CT with fecal samples from CTB-immunized mice resulted in significantly less signal compared to samples collected from PBS immunized control group, with inhibition rates between 20–60%, indicating the neutralization of the holotoxin by e- and pCTB-induced intestinal antibodies. The results suggested that the plant-made aglycosylated B subunit has similar if not better efficacy in comparison with the bacteria-derived original protein. Likewise, serum samples collected from bacterial or plant-produced CTB-vaccinated mice prevented the binding of CT to GM1-ganglioside in a dose dependent manner, with both e- and pCTB showing comparable efficacy strengths (Fig. 7B).

**Figure 8:**
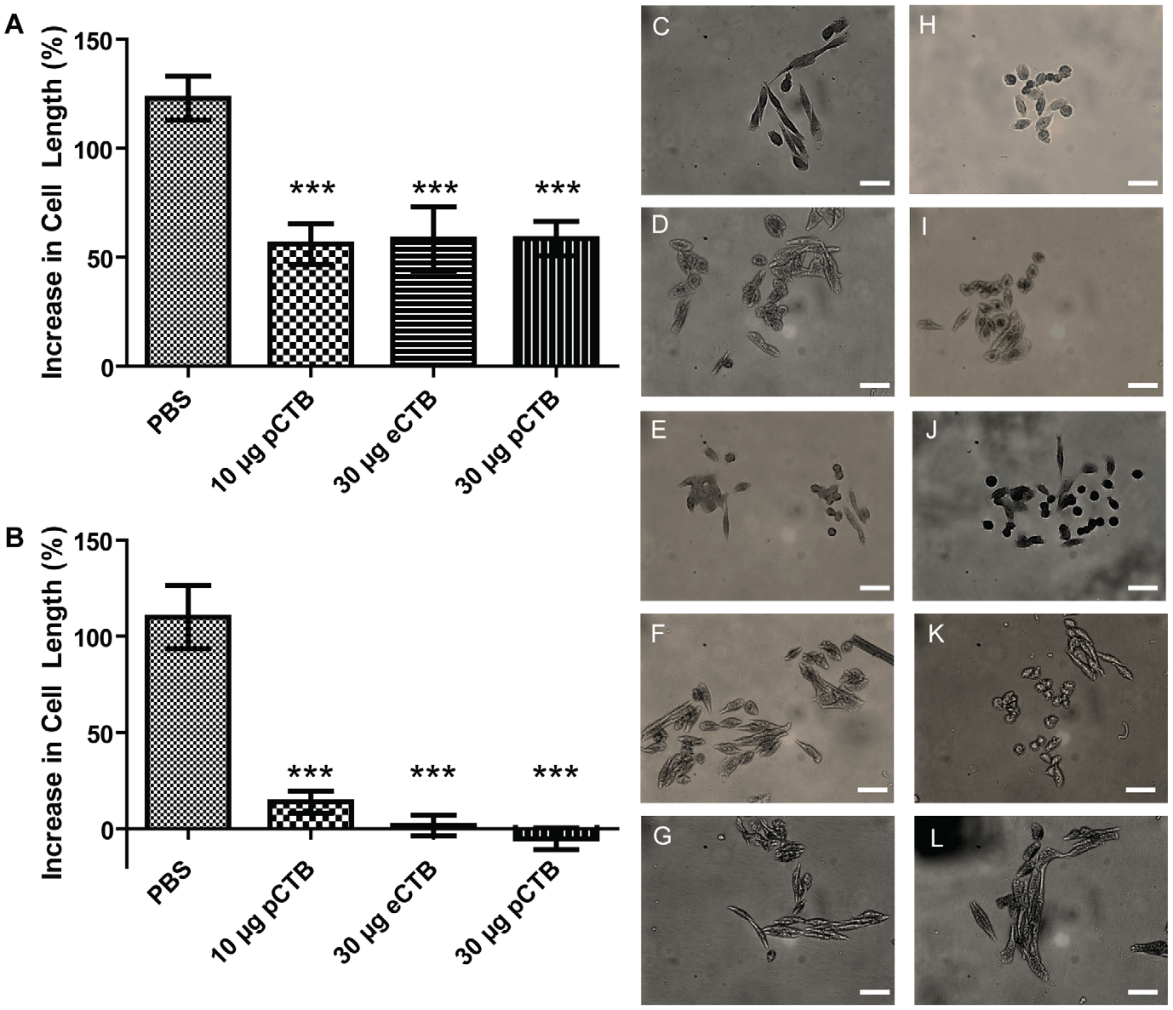
Neutralization of CT holotoxin by pCTB-induced antibodies in the CHO cell elongation assay. *A* and *B*, quantitation of the CHO cell elongation by CT holotoxin pre-treatment with 10× diluted (*A*) fecal and (*B*) serum Ig samples. The holotoxin (20 ng/ml) was pre-incubated with 10× diluted fecal or serum samples obtained 2 weeks after the second vaccination from animals immunized with PBS, 10 or 30 µg pCTB, or 30 µg eCTB. Cell length was measured after overnight incubation and crystal violet staining. See Experimental Procedure for the detailed method. Data are shown as mean ± SEM of triplicate. For both fecal and serum Igs, all immunized groups showed significant neutralization of CT-induced cell elongation compared to the PBS control group: ***P<0.001 (ANOVA with Bonferroni's multiple comparison test). *C*, cells treated with CT. *D*–*G*, cells treated with CT and fecal samples from animals immunized with 10 µg pCTB, 30 µg eCTB, 30 µg pCTB, and PBS, respectively. *H*, untreated control cells. *I*–*L*, cells treated with CT and serum samples from animals immunized with 10 µg pCTB, 30 µg eCTB, 30 µg pCTB, and PBS, respectively. *Scale bar*, 100 µm.

The CHO cell elongation assay [50] was also employed to assess the inhibitory potential of p- and eCTB-induced antibodies. As shown in Fig. 8A, fecal samples from p- and eCTB-treated animals inhibited the CHO cell elongation in a significant manner compared with the extracts obtained from the PBS control group. However, the inhibition was not complete (Fig. 8A, D, E, F, and H), suggesting the partial efficacy at this dilution (10×) of fecal samples. There was no statistically significant difference across groups, e- or pCTB (Fig. 8A, D–F, and data not shown). Higher concentrations of fecal extract samples could not be analyzed due to cell toxicity. By contrast, all serum samples from animals immunized with eCTB, 30 µg, or pCTB, 10 or 30 µg completely abolished the CHO elongation effect of CT (Fig. 8B, H–K). Such high efficacy of serum samples is likely attributed to the higher concentrations of serum Igs than those in fecal samples. As expected fecal extracts and serum samples obtained from PBS immunized mice did not inhibit cell elongation in CHO culture since treatment with these samples lead to a similar cell length compared to the cells incubated in presence of CT only (Fig. 8C, G, and L). Collectively, above results provided compelling evidence that plant-made aglycosylated pCTB can induce holotoxin-neutralizing antibodies upon oral immunization, and this effect is comparable to that of the bacterially produced original B subunit.

## Discussion

In this report, we aimed at developing a robust whole-plant biomanufacturing system for CTB towards economical commercial production to aid in mass vaccination programs. Our initial study revealed *N*-glycosylation of CTB in transgenic *N. benthamiana*. Since such a modification may compromise the protein's safety and efficacy, we created an aglycosylated CTB variant, which was overexpressed using a tobamoviral vector and analyzed the protein in depth for the integrity and feasibility as a vaccine antigen.

The detailed glycan analysis of transgenic *N. benthamiana*-expressed CTB showed a unique glyco-profile containing both high-mannose- and complex-type glycans (Fig. 1 and Table 1). This suggested the protein's broad distribution throughout the endomembrane system in plant cells, albeit with the C-terminal ER retention signal (i.e., KDEL). The proportion of high-mannose-type glycans (16%) was significantly different from that of CTB-MPR expressed in transgenic *N. benthamiana* in our previous study (>75%) [30]. The discrepancy may be partly explained by the efficiency of KDEL receptor's capturing capacity, because the MPR-fused protein had a longer stretch (36 amino acids) on the C-terminus that might have facilitated the receptor's tetrapeptide recognition. Alternatively, as shown in Fig. 3C, a significant fraction of pCTB was shown to have lost the C-terminal Leu or Asp-Leu residues, which might explain the unexpectedly high variability of *N*-glycans of the transgenic plant-expressed CTB molecule. Nevertheless, these two studies clearly showed that *Nicotiana*-expressed CTB displays a significant proportion of Xyl and Fuc-containing glycoforms that could cause potential allergenicity upon clinical use [36]. This provided us with a compelling reason to develop an aglycosylated form of CTB (although the allergenicity concern deserves scientific validation because not all Xyl and Fuc-containing glycoproteins may be allergenic and, in fact, the human mucosal surfaces are constantly exposed to plant glycans via diet and environmental factors).

The initial attempt to express an aglycosylated B subunit in plants using a tobamoviral vector-based overexpression system yielded a low level of the protein, leading us to modify the native *V. cholerae* signal peptide. Recent studies have demonstrated that different secretory signals allow for the accumulation of altered amounts of protein in plant systems [60], [61] and in *Bacillus* systems [62]. For example, plant expressed hemagglutinin levels were tested in combination with cytosolic, chloroplastic, and five different apoplastic modules, with the tobacco calreticulin secretory signal yielding the highest expression level while neither the cytosolic nor chloroplast-targeted hemagglutinin was detected [61]. Herein, we tested expression of pCTB with 12 different apoplastic targeting secretory signals, finding accumulation levels increased up to 40-fold compared to the protein carrying the original *V. cholerae* signal sequence, with rice α-amylase signal peptide consistently giving the highest expression. We investigated if there was a correlation between pCTB expression levels and signal peptide features such as *Nicotiana*-codon adaptation index (CAI; determined by GenScript rare codon analysis tool) [63], theoretical isoelectric point (pI; determined by ExPASy ProtParam tool) [64], and solubility (Grand average of hydropathicity; determined by ExPASy ProtParam tool) [64]; however no linear correlation was found. While the mechanism by which some signal peptides provided a better accumulation of pCTB than others remains elusive, our observations herein and previous similar data on hemagglutinin [61] strongly suggest that the choice of signal peptide has a significant impact on the yield of recombinant proteins in plants. Our results also demonstrated that *N*-glycosylation is not necessary for the high-level production of CTB in plants, despite that in eukaryotic cells *N*-glycans play an important role in the proper folding of newly synthesized polypeptides and help to secure the efficiency of recombinant protein production [32].

GM1-binding is considered to be the most important property responsible for the immunogenicity of CTB. Our data clearly demonstrated that pCTB had analogous IC_50_ values, hemagglutination pattern, and *K*_d_ values to those of native CTB. In accordance to these findings, we demonstrated that e- and pCTB bind to RAW264.7 cells with similar strength (Fig. 4 C and D). It should be mentioned that the SPR sensorgrams for each CTB showed higher experimental R_max_ values than the calculated R_max_. The high experimental R_max_, is hypothesized to be contributed to micelle formation of GM1-ganglioside given that the higher concentrations are acting as a mixed species (soluble+micelle GM1). To address this issue, analysis was performed with GM1 pentasaccharide [65], which does not form micelles, unlike GM1-ganglioside. With GM1 pentasaccharide the experimental R_max_ values were not greater than the calculated R_max_, in addition equilibrium was reached (data not shown).

Conformational stability could have an impact on the protein's biological activities. To this end, pCTB and eCTB had *T*_m_ of >70°C therefore showing good thermal stability. In terms of pH, above pH 5, both CTBs had good thermal stability, and our results showing CTB's low stability below pH 4 were expected due to previous studies demonstrating that CTB pentamers disassemble below pH 3.9 [66]. Our preliminary analysis using SF-HPLC showed that purified pCTB formulated at 1 mg/ml in PBS or in 0.35 M sodium bicarbonate used to neutralize stomach acid remains stable over a month (data not shown), providing an implication for an optimized formulation of pCTB for storage, delivery, and administration.

CTB is arguably one of the few molecules known to date that are able to induce strong mucosal immunity [26], [67]. Our results in a mouse oral immunization study show that pCTB is highly immunogenic, similar to the bacterial B subunit. Indeed, oral administration of as little as 10 µg of the CTB molecule induced a massive immune response, both mucosal and systemic (Fig. 6). Of note, although the animals received only an initial vaccine and a boost 2 weeks later, the antibody titers remained high for over 6 months without a significant drop (Fig. 6D). When using killed *V. cholera* with or without CTB in a field trial, it was suggested that 2 doses of the vaccine may be just as effective as 3 doses [2]. Consistent with this, we did not observe any significant difference in the fecal secretory IgA or serum IgG antibody titers in animals after 2 or 3 vaccinations (data not shown).

Based on the above biochemical, biophysical, and immunological data, we concluded that the modifications introduced to pCTB did not significantly compromise the protein's biological activity, and hence pCTB can serve as a viable alternative to the original B subunit.

For cholera prevention, the role of CTB in the two-component Dukoral vaccine is not fully understood [4], and it is quite possible that by itself, a CTB single component vaccine may not yield effective protection against cholera. However, the protective immunogenicity of CTB in the double-component cholera vaccine has been proven in the Bangladesh large scale field trial [2], [68]. Given the facts that cholera holotoxin is the virulence factor responsible forsevere diarrhea [9] and CTB induces the holotoxin-neutralizing antibodies as shown by others [69], [70], [71] and herein, it is plausible that the protein may help reduce the mortality of cholera under an appropriate immunization condition. Since lowering fatality should be the first priority during outbreaks, it may be worth thoroughly evaluating the effect of CTB as a component of vaccines in reactive immunization programs. The new possibility opened here with the virus-based rapid pCTB production and scalability in plants may help address the above issue. To this end, contract protein manufacturing in *Nicotiana* plants under Good Manufacturing Practices is currently available (e.g., at Kentucky BioProcessing, Owensboro, KY [55], [72]). For example, a standard 4,500 ft^2^ indoor growth area yields ∼1,000 kg of *N. benthamiana* leaf biomass (Barry Bratcher, personal communication). Thus, this facility would have a capacity to produce 1 kg of pCTB in 5 days based on an expression level of 1 g/kg of fresh leaf material, which accounts for 1 million doses of original CTB included in the Dukoral vaccine. Based on the two-column purification process presented here, pCTB may be manufactured at U.S.$1 or even less per mg, which corresponds to one dose of Dukoral vaccine (Josh Morton and Barry Bratcher at Kentucky BioProcessing, personal communication). The pCTB purification procedure developed herein is efficient, and yet has room for improvement; in fact, the initial IMAC process yielded substantial purity (>95%; data not shown). This indicates that the downstream processing could be made more efficient and cost-effective. Consequently, while the actual yield and cost need to be estimated by a commercial-scale run, plant-based production of pCTB may provide an affordable end product at a scale and speed required to aid in reactive mass vaccination during outbreaks.

In summary, our work describes an efficient production of a functional aglycosylated CTB variant in *N. benthamiana* plants. The newly produced molecule was comparable to original CTB for its physicochemical and GM1-ganglioside-binding properties. Moreover, the pCTB exhibited strong and long-lasting oral immunogenicity, yielding antibodies that effectively neutralized the cholera holotoxin *in vitro.* Because the plant virus-based expression system is rapid and readily scalable, pCTB may supplement the current capacity of cholera vaccine production to aid in mass vaccination against cholera outbreaks.

## Supporting Information

Figure S1 **SF-HPLC-based secondary separation of PA-glycans isolated from transgenic** ***Nicotiana*** **-expressed CTB.** The PA-labeled glycans separated by the initial RP-HPLC (Fig. 1C) were further fractionated by SF-HPLC. The peak number shown in each chromatogram corresponds to that of RP-HPLC in Fig. 1C. Lower case letters in chromatograms represent fractions subsequently analyzed for glycan mass and structure, as illustrated in Fig. S2.
(DOC)

Figure S2 **Structural determination of representative PA-glycans isolated from transgenic** ***Nicotiana*** **-expressed CTB.** Detailed analysis for the three most abundant PA-glycan peaks, i.e., Peak 6-b (*A* and *D*), 1-d (*B* and *E*), and 3-f (*C* and *F*) are shown. Peak numbers correspond to those of SF-HPLC in Fig. S1. *A*–*C*, comparative RP-HPLC chromatograms showing the elution positions of Peak 6-b, 1-d, and 3-f matching those of standard PA-labeled Man_3_Xyl_1_GlcNAc_2_-PA (M3X), Man_3_Xyl_1_Fuc_1_GlcNAc_2_-PA (M3FX), and GlcNAc_1_Man_3_Xyl_1_Fuc_1_GlcNAc_2_-PA (^GN^M3FX), respectively. Note that two possible isomeric forms of GlcNAc_1_Man_3_Xyl_1_Fuc_1_GlcNAc_2_-PA were analyzed in *C*, demonstrating that the glycan of Peak 3-f corresponds to the one with the terminal GlcNAc attached to the α1, 6-linked mannose (^GN^M3FX), but not to the α1, 3-linked mannose (_GN_M3FX). *D*–*F*, MALDI-TOF-MS analysis showing that the molecular masses of the subjects correspond to the theoretical values of RP-HPLC-determined glycan structures.
(DOC)

Figure S3 **Haemagglutination assay.** Performed as previously described (Matoba 2008). Briefly, a 1% solution of sheep red blood cells coated with GM1 ganglioside was incubated overnight at 4°C with the indicated concentrations of e- or pCTB. Haemagglutination was visualized using a Cellular Technology Ltd. ImmunoSpot. Samples were analyzed in duplicate. Native CTB and pCTB displayed a similar haemagglutination pattern.
(DOC)

Table S1 **Secretory signal peptides used for pCTB expression.** Sources of signal peptides are shown along with their corresponding plasmid names and GenBank accession numbers.
(DOC)
