# Supplementary figures and images for: Rapid and Scalable Plant-based Production of a Cholera Toxin B Subunit Variant to Aid in Mass Vaccination against Cholera Outbreaks
Source: PLoS Negl Trop Dis. 2013 Mar 7;7(3):e2046. doi: 10.1371/journal.pntd.0002046 (PMC3591335; doi:10.1371/journal.pntd.0002046)

Figure S1

**
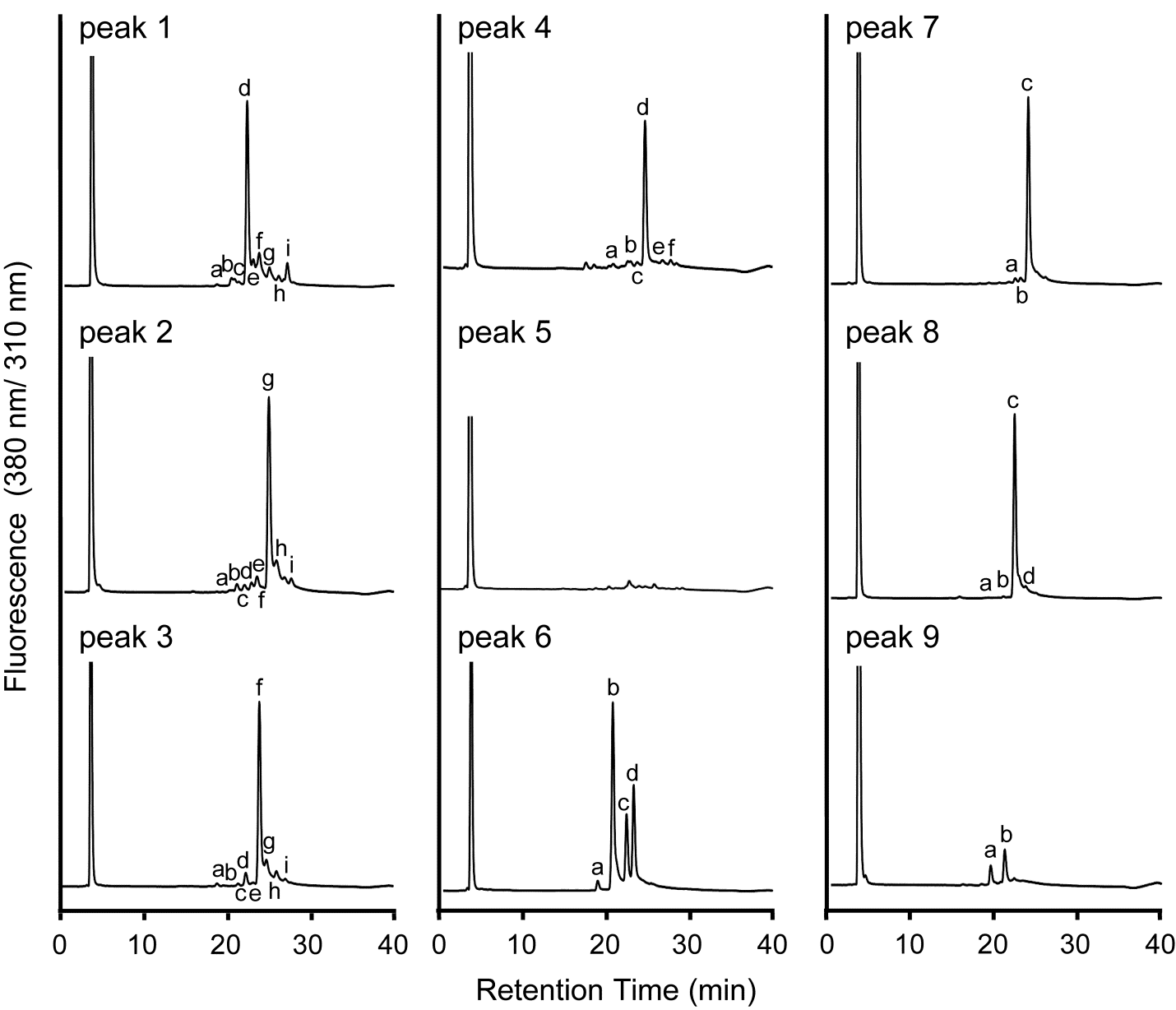
**

Supplement: Figure S1 — SF-HPLC-based secondary separation of PA-glycans isolated from transgenic Nicotiana -expressed CTB. The PA-labeled glycans separated by the initial RP-HPLC (Fig. 1C) were further fractionated by SF-HPLC. The peak number shown in each chromatogram corresponds to that of RP-HPLC in Fig. 1C. Lower case letters in chromatograms represent fractions subsequently analyzed for glycan mass and structure, as illustrated in Fig. S2. (DOC) [file pntd.0002046.s001.doc]

Figure S2

**
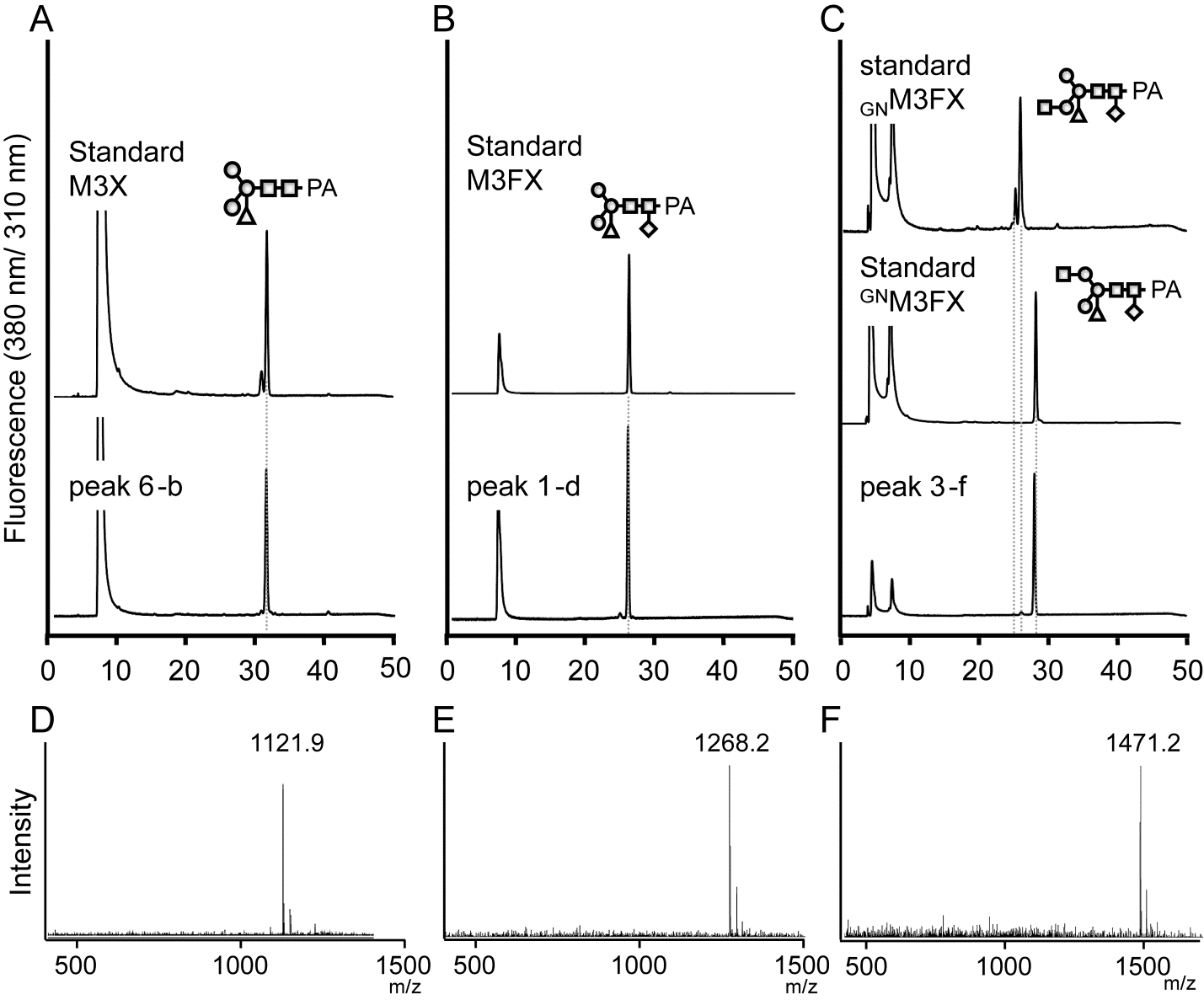
**

Supplement: Figure S2 — Structural determination of representative PA-glycans isolated from transgenic Nicotiana -expressed CTB. Detailed analysis for the three most abundant PA-glycan peaks, i.e., Peak 6-b (A and D), 1-d (B and E), and 3-f (C and F) are shown. Peak numbers correspond to those of SF-HPLC in Fig. S1. A–C, comparative RP-HPLC chromatograms showing the elution positions of Peak 6-b, 1-d, and 3-f matching those of standard PA-labeled Man3Xyl1GlcNAc2-PA (M3X), Man3Xyl1Fuc1GlcNAc2-PA (M3FX), and GlcNAc1Man3Xyl1Fuc1GlcNAc2-PA (GNM3FX), respectively. Note that two possible isomeric forms of GlcNAc1Man3Xyl1Fuc1GlcNAc2-PA were analyzed in C, demonstrating that the glycan of Peak 3-f corresponds to the one with the terminal GlcNAc attached to the α1, 6-linked mannose (GNM3FX), but not to the α1, 3-linked mannose (GNM3FX). D–F, MALDI-TOF-MS analysis showing that the molecular masses of the subjects correspond to the theoretical values of RP-HPLC-determined glycan structures. (DOC) [file pntd.0002046.s002.doc]

Figure S3

**
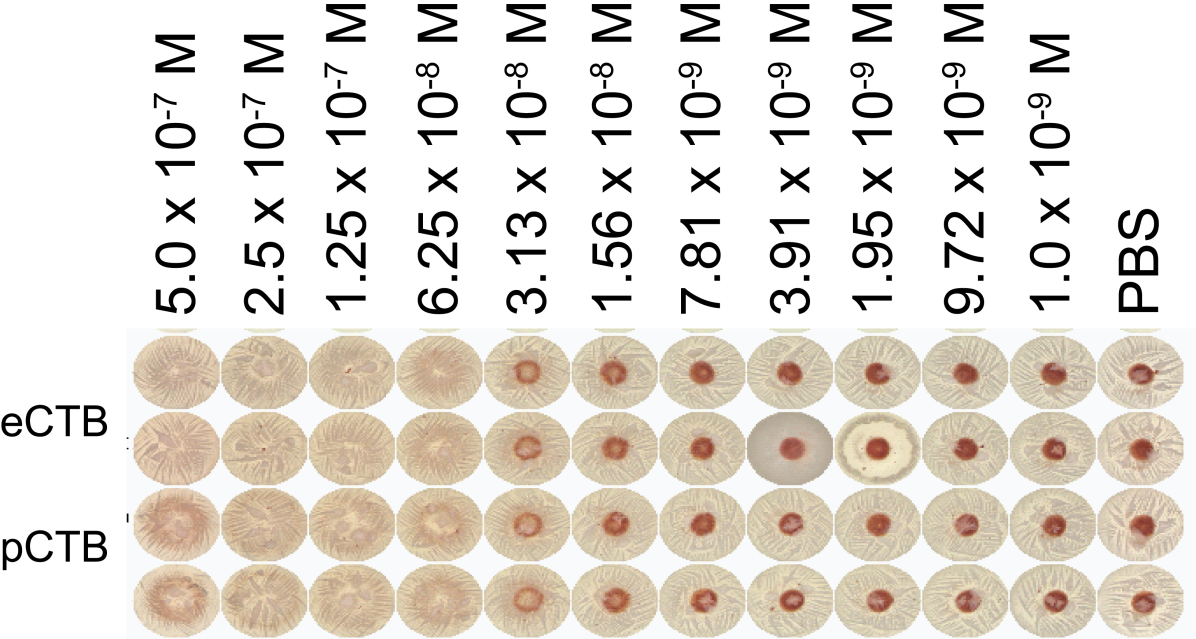
**

Supplement: Figure S3 — Haemagglutination assay. Performed as previously described (Matoba 2008). Briefly, a 1% solution of sheep red blood cells coated with GM1 ganglioside was incubated overnight at 4°C with the indicated concentrations of e- or pCTB. Haemagglutination was visualized using a Cellular Technology Ltd. ImmunoSpot. Samples were analyzed in duplicate. Native CTB and pCTB displayed a similar haemagglutination pattern. (DOC) [file pntd.0002046.s003.doc]
