# Supplementary material for: Rapid and Scalable Plant-based Production of a Cholera Toxin B Subunit Variant to Aid in Mass Vaccination against Cholera Outbreaks
Source: PLoS Negl Trop Dis. 2013 Mar 7;7(3):e2046. doi: 10.1371/journal.pntd.0002046 (PMC3591335; doi:10.1371/journal.pntd.0002046)
Supplement: Table S1 — Secretory signal peptides used for pCTB expression. Sources of signal peptides are shown along with their corresponding plasmid names and GenBank accession numbers. (DOC) [file pntd.0002046.s004.doc]

**Table S1.** Secretory signal peptides used for pCTB expression.

| **Plasmid** | **Signal Peptide Source** | **GenBank Accession Number** |
| --- | --- | --- |
| pNM226  pNM227 | *Hordeum vulgare* chitinase | CAA55344, nucleotides 1 to 78  CAA08910, nucleotides 1 to 72 |
|  | *Solanum tuberosum* Glucan endo-1,3-beta-D-glucosidase |  |
| pNM228 | *Arabidopsis thaliana* Auxin-binding protein 1 | NP_192207, nucleotides 1 to 99 |
| pNM229 | *Pectobacterium atrosepticum* Pel B | Q6CZ3, nucleotides 1 to 66 |
| pNM230 | *Phaseolus vulgaris* endopolygalacturonase-inhibiting protein (PGIP) | P55823, nucleotides 1 to 87 |
| pNM231 | *Nicotiana tabacum* PR1a | ABV21361, nucleotides 1 to 90 |
| pNM232 | *Oryza sativa* Glutelin | P14323, nucleotides 1 to 72 |
| pNM257  pICH20155 | *Vibrio cholerae* cholera toxin | U25679, nucleotides 1 to 63 |
|  | Rice α-amylase | P27932 |
| pICH20188 | *Nicotiana plumbagenifolia* calreticulin | Z71395 |
| pICH20388  pICH20999 | Apple pectinase | P48978 |
|  | Barley α-amylase | CAX51374 |
